# Real-time *in vivo* optogenetic neuromodulation and multielectrode electrophysiologic recording with NeuroRighter

**DOI:** 10.3389/fneng.2014.00040

**Published:** 2014-10-29

**Authors:** Nealen G. Laxpati, Babak Mahmoudi, Claire-Anne Gutekunst, Jonathan P. Newman, Riley Zeller-Townson, Robert E. Gross

**Affiliations:** ^1^Translational Neuroengineering Group, Department of Biomedical Engineering, Georgia Institute of Technology and Emory University School of MedicineAtlanta, GA, USA; ^2^Department of Neurosurgery, Emory University School of MedicineAtlanta, GA, USA; ^3^Picower Institute for Learning and Memory, Massachusetts Institute of TechnologyCambridge, MA, USA; ^4^Laboratory for Neuroengineering, Department of Biomedical Engineering, Georgia Institute of Technology and Emory University School of MedicineAtlanta, GA, USA; ^5^Department of Neurology, Emory University School of MedicineAtlanta, GA, USA

**Keywords:** optogenetics, NeuroRighter, electrophysiology, LED, open-source, closed-loop, microelectrode array

## Abstract

Optogenetic channels have greatly expanded neuroscience’s experimental capabilities, enabling precise genetic targeting and manipulation of neuron subpopulations in awake and behaving animals. However, many barriers to entry remain for this technology – including low-cost and effective hardware for combined optical stimulation and electrophysiologic recording. To address this, we adapted the open-source NeuroRighter multichannel electrophysiology platform for use in awake and behaving rodents in both open and closed-loop stimulation experiments. Here, we present these cost-effective adaptations, including commercially available LED light sources; custom-made optical ferrules; 3D printed ferrule hardware and software to calibrate and standardize output intensity; and modifications to commercially available electrode arrays enabling stimulation proximally and distally to the recording target. We then demonstrate the capabilities and versatility of these adaptations in several open and closed-loop experiments, demonstrate spectrographic methods of analyzing the results, as well as discuss artifacts of stimulation.

## INTRODUCTION

Optogenetic techniques provide powerful tools for precise manipulation of complex nervous system circuitry. Selective excitation and inhibition with light of a genetically targeted neuron population – without directly perturbing the neighboring untargeted cells – has provided the means to elegantly explore a number of important neuroscience questions (Aravanis et al., 2007; Carter et al., 2009; Gradinaru et al., 2009; Kravitz et al., 2010; Yizhar et al., 2011; Packer et al., 2012; Wykes et al., 2012; Paz et al., 2013). When combined with electrophysiological recording techniques, optogenetic control can provide unprecedented insight into neural connectivity and function (Bell et al., 2013), as well as suggest potential therapeutic strategies (Gradinaru et al., 2009; Paz et al., 2012; Wykes et al., 2012; Krook-Magnuson et al., 2013).

Optogenetics combines a number of techniques in molecular biology, electrophysiology, optics, and neuroscience, the mastery of which can prove a barrier to easy adoption. Significant efforts have been made to expand the toolbox of optogenetic channels, constructs, and viral techniques (Chow et al., 2010; Gunaydin et al., 2010; Diester et al., 2011), as well as to develop complex custom-designed optoelectric neural interfaces (Fan et al., 2013; Voigts et al., 2013). However, commercial electrophysiology hardware and software has lagged behind these developments, and often fails to incorporate support for complex stimuli, real-time multielectrode closed-loop control (Newman et al., 2013), and customized experimental configurations in awake and behaving animals. In addition, the cost of these systems is often prohibitive, particularly for investigators looking to initiate a new line of research with limited funding. Custom systems have been developed and reported previously (Armstrong et al., 2013; Nguyen et al., 2014), but have been designed for a particular narrow focus. As a result they can be limited in their customizability and application to any particular experiment, particularly in regard to stimulation parameters and patterns. The price of setting up one of these custom systems may also be prohibitive, particularly if they use high-quality lasers for stimulation. There is consequently a need for a customizable, adaptive, and low-cost optoelectrophysiology system for *in vivo* experimentation.

### NEURORIGHTER PLATFORM

We developed our optoelectrophysiology platform based on the existing hardware and software for electrical stimulation and electrophysiology, NeuroRighter. NeuroRighter is a low-cost open-source electrophysiology system written in C-sharp and intended for open and closed-loop neural interfacing *in vivo* and *in vitro* (Rolston et al., 2009b,c, 2010a). The software, compatible with 32- and 64-bit Windows operating systems (Microsoft Corporation, Redmond, WA, USA) is free and the source code is available on a publicly accessible repository^1^. The hardware is also open-source, utilizing printed circuit boards (PCBs) and commercially available components, National Instruments (NI; National Instruments Corporation, Austin, TX, USA) data acquisition hardware (NI PCI-6259, PCI2-6259, PCI2-6353, and PCIe-6363 16-bit 1 M sample/sec) and driven with NI’s hardware control library, DAQmx. The design, construction, and performance of this electrophysiology platform – which meets or exceeds the performance of many commercial alternatives – is well documented (Rolston et al., 2009c; Newman et al., 2013).

**FIGURE 1:**
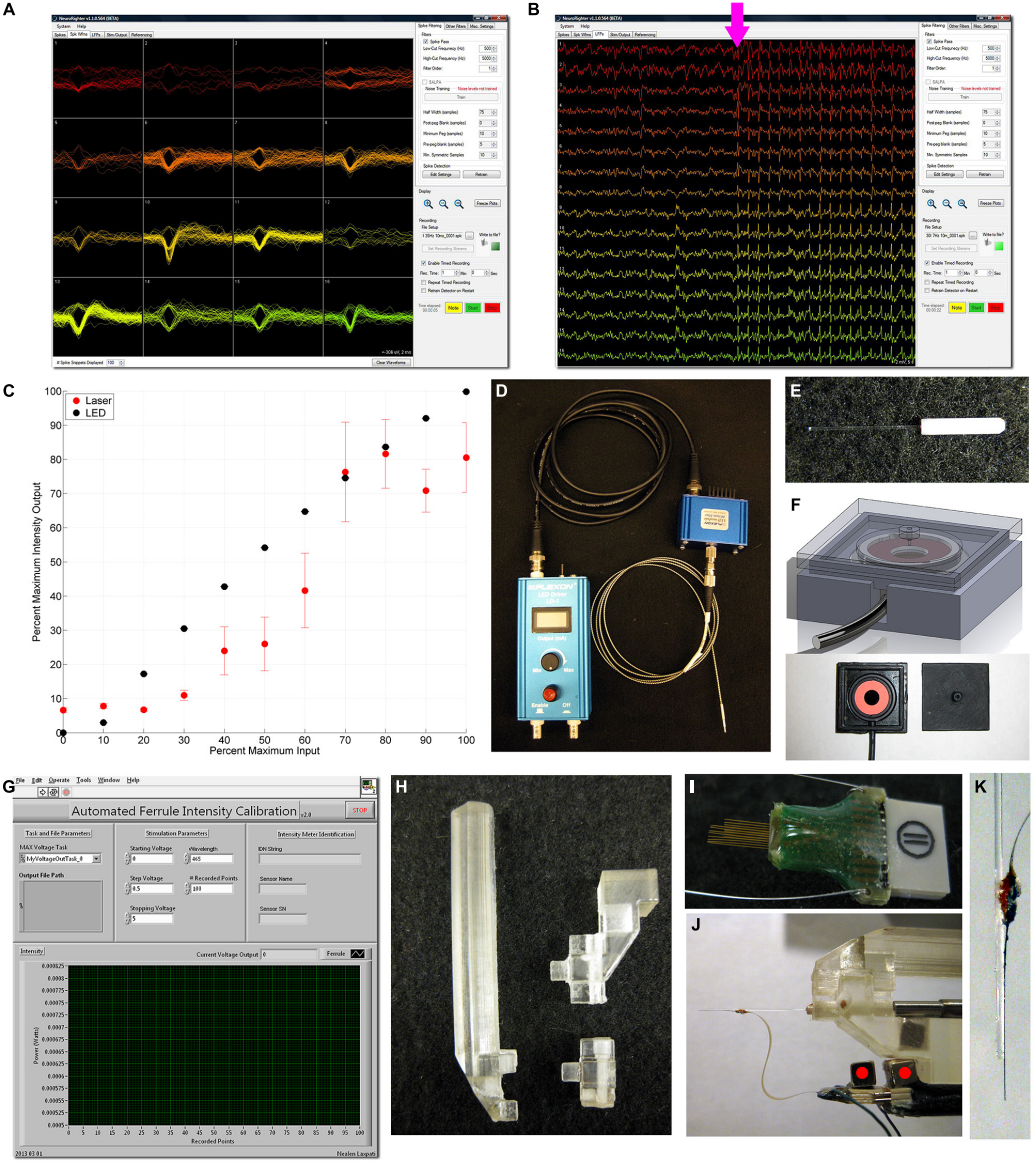
**NeuroRighter software and hardware for calibration, optical stimulation, and recording.** NeuroRighter’s main application window enables real-time isolation of single units **(A)** and local field potentials (LFP; **B**) from multielectrode arrays, with real-time visualization of the electrophysiologic response to optical stimulus (**B**, magenta arrow). **(C)** A comparison between the mean (dot) and SD (error bars) normalized output of a 473 nm blue DPSS Laser (Shanghai Dream Laser; red) and 465 nm blue LED (Plexon, Inc.) output (black). Note the high variance associated with the laser output as compared to the LED (present for the LED but smaller than the data point marker), and the non-linear nature of the laser output. **(D)** Plexon LED Driver, 465 nm blue LED module, and 200 μm 0.67 NA armored patch fiber cable. **(E)** Custom-made optical ferrules utilizing 200 μm diameter 0.37 NA fiber optic. **(F)** 3D-printed Intensity Chamber design (top) and fabricated with accompanying photodiode (bottom). Custom-designed and fabricated ferrules **(E)** plug into the chamber (**F**, bottom right) which overlays the S121C detector (**F**, bottom left). **(G)** Labview-based program for Automated Ferrule Intensity Calibration **(C)**.** (H)** A 3D-printed implantation post has multiple modules to enable ferrule implantation alone (**H**, bottom right) or coupled to an array **(H**, top right,** J)**. **(I)** TDT 16-channel MEA, which was implanted in the dorsal hippocampus targeting CA1 and CA3 simultaneously. **(J)** A H-style NeuroNexus 16 channel shank array was manually glued to a calibrated ferrule **(K)**, enabling simultaneous stimulation, and recording from the same target site.

Recently, the NeuroRighter platform has been enhanced for improved usage with closed-loop multichannel interfacing experiments for electrical stimulation (Newman et al., 2013), as well as *in vitro* optogenetic stimulation (Tchumatchenko et al., 2013). NeuroRighter is capable of recording single-unit (**Figure 1A**) and local field potential (LFP; **Figure 1B**) activity from multielectrode extracellular arrays, as well as delivering complex and customizable patterns of electrical stimulation through analog and digital outputs (Rolston et al., 2009c, 2010a; Newman et al., 2013). NeuroRighter is consequently well-positioned to incorporate customized optogenetic hardware and provide a low-cost solution to the problems facing optoelectrophysiology.

Here, we summarize the adaptations we have made to NeuroRighter to produce a system that enables real-time optogenetic neuromodulation and multielectrode electrophysiology *in vivo* in awake and behaving rodents using low-cost components. We describe two example experiments, one in which the site of optical stimulation is distant from the electrode recordings (medial septum (MS) and dorsal hippocampus, respectively), and the other in which optical stimulation and electrophysiologic recording is performed in the same location (dorsal hippocampus). In the former, we provide examples of the complex stimuli that can be performed with NeuroRighter, and present descriptive results. In the latter, we demonstrate and discuss some of the issues concerning optically induced artifacts.

## DESIGN

### DESIGN CRITERIA

We designed our optoelectrophysiology system to adapt the *in vivo* capabilities of NeuroRighter into the optogenetic purview. In so doing, we wished to maintain the standards established in its original design – that the system be (1) inexpensive, interfacing with commercially available hardware as well as custom-designed solutions; (2) maintain the high spatial and temporal resolution required in electrophysiology; (3) function robustly in a number of different experimental environments; and (4) be open-source.

### HARDWARE AND SOFTWARE FOR OPTICAL STIMULATION

While many of efforts with optogenetics relied on the use of lasers (Yizhar et al., 2011; Armstrong et al., 2013), high-intensity light-emitting diodes (LEDs) have increasingly proven an attractive alternative, particularly for *in vivo* experiments (Cardin, 2012; Nguyen et al., 2014). Lasers tend to be large and cumbersome, and many setups require careful collimation and alignment for proper function and maintenance of consistent output within and between experiments. These designs are sensitive to the slight perturbations generated from connections to awake and behaving animals. Collimated LEDs, however, are compact, robust, and readily portable, making them easy to integrate into behavioral experiments. In addition, LEDs have a more precise input/output relationship than similarly-priced lasers. LED luminance output can be well approximated by a logarithmic or linear function with respect to input current. In contrast, similarly priced DPSS lasers have a non-linear sigmoidal relationship with input voltage (**Figure 1C**; Cardin, 2012). Furthermore, the light intensity generated by these lasers can be unstable and demonstrate transient peaks and fluctuations (Cardin, 2012). The output intensity of LEDs, in contrast, is much more stable and better approximates a square wave, with much less variation over time. Indeed, we have determined that the variability in 465 nm Blue LED output intensity is less than that of a comparable-cost laser 475 nm DPSS Laser (Shanghai Dream Lasers, China; **Figure 1C**). While the standard deviation of the laser intensity output could be over 10% of the maximum output, the standard deviation of the LED intensity output was small enough to be obscured by the datapoint marker. It should be noted that the outputs of lasers and LEDs are influenced by temperature as well. Without proper heat dissipation, output efficiency will decrease and consistency will no longer be maintained (Newman, 2013). Controller properties also largely influence these input dynamics: more advanced and more expensive controllers can linearize laser outputs, in particular when coupled with optical feedback. Indeed, for experiments with both LEDs and lasers in which long-term stimulation may warrant heat dissipation, it is recommended that an optical feedback controller be used to maintain consistency in optical stimulation output. High-intensity LEDs enable precise experimental standardization and repeatability while also retaining the high-intensity output and dynamic range that make lasers desirable for optogenetic experiments. Consequently, we designed our platform to make use of low-cost high intensity LEDs in optogenetic *in vivo* experiments in awake and behaving animals.

To this end, we made use of commercially available high-intensity LEDs (Plexon Inc., Dallas, TX, USA; **Figure 1D**). Similar LEDs are available from other suppliers (Thorlabs, Newton, NJ, USA), and the cost of these is in a similar price range (∼$2000 total with current driver), which makes the cost of the total NeuroRighter system with optogenetics about $12,000. The 465 nm blue LED was controlled by a voltage-to-current controller (Plexon Inc.), and output light along a patch fiber cable connected via FC/PC connection. The LED controller received input from one channel of the analog output from a NI SCB-68 screw-terminal connector box. This output ranged from 0 to 5 V, which was converted by the controller to 0–300 mA of current. This system was capable of driving 465 nm Blue LED light output at intensities of up to 80 mW/mm^2^ in custom-made implantable optical ferrules (**Figure 1E**) – well within the acceptable window for non-damaging optical stimulation (Cardin et al., 2010). As each analog output of NeuroRighter can be accessed independently, four LEDs can be simultaneously controlled with NeuroRighter configuration on a single supported NI data acquisition card. The modular nature of the system enables the addition of additional NI data acquisition cards to increase the number of LED outputs, in addition to recording inputs.

Custom-made implantable optical ferrules (**Figure 1E**) were constructed from 1.25 mm long 230 μm inner diameter ceramic stick ferrules (Precision Fiber Products, Milpitas, CA, USA) in a fashion based on a previously well-described design (Sparta et al., 2012). 200 μm diameter 0.37 numerical aperture optical fiber (Thorlabs) was carefully stripped of its protective coating and cleaved. Heat-cure epoxy (Precision-Fiber Products) was mixed and applied to the concave end of the ferrule, through which the cleaved fiber segment was subsequently threaded. After wiping off the excess, a heat gun was applied to quickly cure the epoxy, and the ferrules were then allowed to finish curing overnight at room temperature. The ferrule connector was polished using a polishing disk and increasingly fine grades of polishing paper (Thorlabs), with frequent inspection to ensure transmission quality. Once polished, the free end of the fiber was scored and cleaved to 10–12 mm in length.

Custom hardware and software was designed in order to standardize the variations in output intensity and calibrate each ferrule^2^. An intensity calibration device (ICD; **Figure 1F**, bottom) was designed in Solidworks 2011 (Dassault Systèms Solidworks), 3D-printed on an Objet Eden 250 from FullCure 720 model resin, and painted black. A S121C silicone diode (Thorlabs) was placed within the central cavity of the ICD and connected to a PM100USB intensity meter (**Figure 1F**, top). Custom-written LabVIEW 2009 software (National Instruments, Austin, TX, USA; **Figure 1G**) steps the LED through user-defined output voltages and measures the resultant power for a defined wavelength and number of points on the S121C silicone diode. LED output power passing through the ferrule is thus correlated to the analog input voltage signal to the LED controller. The program then calculated intensity from power based on the diameter of the fiber optic and linearly correlated to the voltage input. This standardized the output of each ferrule based on intensity rather than voltage input, enabling precise stimulation at accurate intensities across all experimental subjects. Custom-written Matlab scripts then converted standard output intensities to the appropriate signal voltages for each test subject.

Ferrules were attached to the patch fiber cable by means of 1.25 mm inner diameter ceramic split sleeves (Precision Fiber Products). These were reinforced by threading them through trimmed heat shrink tubing (Digi-Key, Thief River Falls, MN, USA), and subsequently heating them. These reinforced sleeves were superior to the bare split-sleeves in resisting breakage due to vigorous movement of some subjects. This ceramic split sleeve was the most common breaking point in the connection, conveniently leaving the implanted ferrule and patch fiber cables intact.

### ELECTRODE ARRAYS

Two electrode array configurations were used in these proof-of-concept experiments. For recording of the dorsal hippocampus while simultaneously stimulating the MS, 16-channel microwire multielectrode arrays [Tucker Davis Technologies (TDT), Alachua, FL., USA; MEA] were constructed from sixteen 33 μm diameter tungsten electrodes with polyimide insulation (**Figure 1I**). The electrodes were arranged in two rows of eight electrodes with 1 mm between rows and 175 μm of space between the electrodes within a row. Ground and reference wires were separated on the array and routed through two stainless steel wires, which were affixed to separate skull screws during the implantation surgery. The two rows were cut to different lengths, 4.0 and 3.0 mm, to target and record simultaneously from the hippocampal CA3 and CA1 regions, respectively, enabling multiunit and LFP recording from the hippocampus distantly from the optical stimulation site in the MS.

NeuroNexus (Ann Arbor, Michigan, USA) 16-channel shank arrays were coupled with optical ferrules to record and stimulate simultaneously in the hippocampus. A single-shank H-style array was used, with 16 177 μm^2^ contacts spaced 100 μm apart along a 5 mm shaft. This length was sufficient to record simultaneously from the CA1 and CA3 layers. The shaft was connected to an Omnetics connector via a 21 mm flexible ribbon cable. Ground and reference wires were again separated from the contact sites and routed through stainless steel wires. NeuroNexus “activated” the electrode contacts via iridium oxide – a process that reduced impedance and they suggested would reduce optical stimulation artifacts (personal communication). Both the NeuroNexus and TDT arrays made use of a magnet-based coupling technique to the 16-channel 100 gain tethered recording headstage (Triangle Biosystems, Durham, NC, USA) to reduce movement artifacts (**Figure 1J**, red dots), a technique we have described previously (Rolston et al., 2009c, 2010b). Once the magnet was attached with superglue, the NeuroNexus array could be situated onto our custom-designed and 3D-printed implantation holder^3^ (**Figures 1H,J**). This enabled the array shank and contacts to be positioned in parallel to the optical fiber (**Figure 1J**), and cemented in place with quick-drying super glue (**Figure 1K**). The fiber and shank thus were stereotactically inserted together, maintaining a fixed distance from each other throughout the experiment.

The implantation device consists of a single post compatible with a Kopf Universal Holder (David Kopf Instruments, Tujunga, CA, USA) with a single-prong plug that enabled easy swapping and customization depending on the implant configuration (**Figure 1H**). This allowed us to use the device to implant an optical ferrule in isolation – as in the MS – or in conjunction with a NeuroNexus array (**Figure 1J**) – as in the dorsal hippocampus.

## EXPERIMENTAL METHODS

### SURGERIES

Two month old adult male Sprague–Dawley rats (250–300 g) were purchased from Charles River Laboratories (Wilmington, MA, USA). All animals were maintained within a 12/12 light/dark cycle vivarium with unlimited access to food and water. This work was conducted in accordance with Emory University’s Institute for Animal Care and Use Committee.

Each subject underwent two surgical procedures. The first survival surgery introduced the optogenetic viral vector to the stimulation target – either the MS or the dorsal hippocampus. For medial septal stimulation, rats were anesthetized with 1.5–4% inhaled isoflurane, and a craniectomy was made 0.40 mm anterior and 2.00 mm lateral to bregma on the right side of the skull. A pulled-glass pipette attached to a stereotactically mounted injector (Nanoject; Drummond Scientific Co., Broomall, PA, USA) was used to inject 1.8 μL of 10^12^ particles/mL AAV5-hSynapsin-hChR2(H134R)-EYFP (UNC Vector Core Services, Chapel Hill, NC, USA). AAV5-hSynapsin-EYFP (UNC Vector Core Services) was used in control animals. The injection was made at a 20∘ angle to the dorsal-ventral axis (0.40 mm anterior, 2.12 mm lateral at the 20∘ angle, 5.80 mm ventral to pia along the rotated axis) in order to target the MS without damaging the medially located central sinus. After 5 min of equilibration the injection was made over 7 min with the pipette remaining in place an additional 10 min post-injection to prevent reflux. Once withdrawn, the scalp was stapled closed, ketofen was administered as an analgesic (3–5 mg/kg) to minimize pain, and the rats were quarantined for 72 h before returning to normal housing. Hippocampal injections were similarly performed, but the craniectomy was made 3.30 mm posterior and 3.20 mm lateral over the right dorsal hippocampus. An injection of 1.8 μL of 10^12^ particles/mL AAV2-CaMKIIα-hChR2(H134R)-mCherry was made along the dorsal–ventral axis at 3.10 mm depth to pia to target the hippocampal pyramidal neurons. Identical closure and quarantine procedures were performed.

The second survival surgery was performed two weeks later, which we have found to provide ample time for robust channel expression. For the medial septal stimulation experiments, a second craniectomy was made over the right dorsal hippocampus centered at 3.50 mm posterior and 2.80 mm lateral to bregma. The dura was incised with a sterile curved scalpel blade. The TDT array was positioned at a 50∘ angle to midline, with the posterior end swung laterally, to match the positioning of the hippocampal pyramidal cell layers (Rolston et al., 2010b). The MEA was lowered while simultaneously recording single unit and LFP activity to attain the ideal positioning (Rolston et al., 2009b). When the electrophysiologic recordings stabilized, the original injection craniectomy was reopened, and a calibrated optical fiber ferrule was implanted at a 20∘ angle to the dorsal–ventral axis (0.40 mm anterior, 2.12 mm lateral in the rotated axis). Stimulation was performed as the ferrule was implanted, with the resulting recordings immediately analyzed spectrographically. Descent was halted when a strong stimulus-response signal was observed in the spectrogram, or when the optical ferrule reached a depth of 5.50 mm from pia along the rotated axis.

For the hippocampal stimulation experiment, the previous craniectomy was reopened and expanded, and the combined optical fiber and NeuroNexus electrode array (**Figure 1J**) was inserted while similarly stimulating. Stimulation artifacts were noted in the upper cortical layers where there was no viral expression, and were recorded for later artifact analysis. A LFP response was visible in the hippocampus in addition to the artifact and so the implantation was halted at 2.80 mm at the shank tip. In both experiments, once the electrodes and ferrules were in place, the craniectomy was sealed with dental acrylic (OrthoJet; Lang Dental; Wheeling, IL, USA), securing the array/electrode and ferrule in place. The rats were administered ketofen (3–5 mg/kg) to minimize pain and returned to normal housing to recover for 3–5 days.

### OPTICAL STIMULATION AND ELECTROPHYSIOLOGIC RECORDINGS

Using our adapted NeuroRighter system, electrophysiologic recordings were sampled at 25 kHz with a 1–9,000 Hz bandwidth. LFPs were isolated online with a 1–500 Hz 1-pole Butterworth band-pass filter and downsampled to 2000 Hz. Action potentials were isolated both online (Newman et al., 2013) and oﬄine, with the oﬄine results presented here. Action potentials were detected oﬄine using custom-written adaptations to the automated spike-sorting Wave_clus scripts (Quiroga et al., 2004). The raw data was band-pass filtered oﬄine from 500 to 5000 Hz. For the TDT electrodes, the median signal was removed across the CA3 and CA1 electrodes, respectively. For the NeuroNexus Array, the median signal was removed across all electrodes. Positive and negative thresholds were applied at 5x the SD of the signal, and the resulting waveforms were matched, sorted, and isolated using superparamagnetic clustering (Wave_clus; Quiroga et al., 2004). Power spectra and spectrograms were computed using the Chronux suite of analysis tools and multitaper analysis (Bokil et al., 2010), with a moving window size of 4 s stepping in 0.5 s increments, *T* = 4, *W* = 1, and seven tapers. Data were recorded intraoperatively and for up to 4 weeks postoperatively.

To stimulate awake and behaving animals, calibrated ferrules were connected via armored patch fiber cables (200 μm diameter, 0.67 NA, Plexon). Square-wave stimulation pulses varied between 10, 30, and 50 mW/mm^2^; 7, 11 (theta), 17, 23, 35 (beta), and 42 (gamma) Hz; and 2, 5, and 10 ms pulse widths. NeuroRighter enables custom-designed stimulation times and amplitudes to be defined via Matlab script (Newman et al., 2013). We leveraged this customizability to develop several other stimulation patterns, including varying frequency, Poisson distributions, and continuous sinusoids, which are described in more detail as they are presented. In all cases, the experimental protocol consisted of repeated 1 min recordings of 20 s of background, 20 s of stimulation with a particular pattern, and a subsequent 20 s of additional background. Stimulation protocols were performed in random order and repeated numerous times over several recording sessions. This setup was able to stimulate and record LFP and single-unit responses from awake and behaving animals uninterrupted for several hours and over several days.

### HISTOLOGY

Histology was performed after experimentation to verify microelectrode recording locations and light-sensitive ion channel expression. Rats were deeply anesthetized with an overdose of Euthasol (5 ml/kg, Virbac, Fort Worth, TX, USA) injected intraperitoneally. They were then transcardially perfused with 0.9% saline followed by 4% paraformaldehyde in 0.1 M phosphate buffer at pH 7.2. The heads, still containing the electrodes and ferrules, were then separated and post-fixed at 4∘C overnight. The next day, the brains were dissected out, removed, and cryoprotected with 30% sucrose at 4∘C. Frozen transverse (horizontal) sections were made of 50 μm thickness on a sliding microtome and collected in 0.1 M PBS. Sections were mounted on glass slides and mounted with Vectashield mounting medium with DAPI (Burlingame, CA, USA) for visualization of nuclei. Sections were imaged in the NIS-Elements software (Nikon Instruments, Inc., Melville, NY, USA) using a Nikon DS-Fil color digital camera on a Nikon E400 microscope equipped with TRITC, FITC, and DAPI fluorescence cubes.

## RESULTS

### HISTOLOGIC VALIDATION OF CHANNEL EXPRESSION AND ELECTRODE PLACEMENT

**FIGURE 2:**
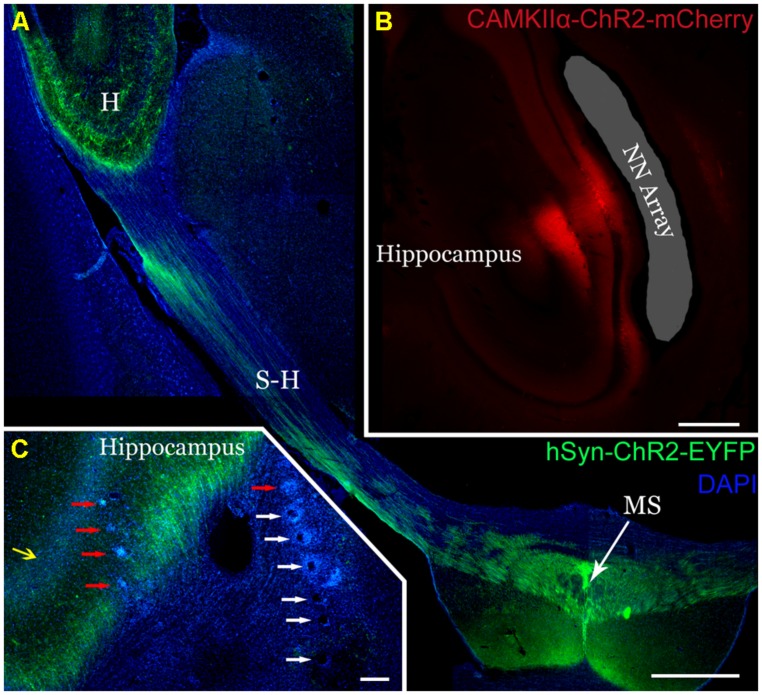
**Robust expression of ChR2 on transverse section histology and verification of electrode placement. (A)** AAV5-hSyn-ChR2-EYFP injection into the medial septum (MS) produced robust ChR2-EYFP expression (green). Axons from the MS express along the septohippocampal pathway (S-H) and into the hippocampus (H). Scale 1 mm. **(B)** Similarly, injection of AAV2-CaMKIIα-ChR2-mCherry into the dorsal hippocampus (red) selectively expressed in the pyramidal cell layer and their projecting axons/dendrites. The NeuroNexus array implantation site was localized alongside the pyramidal cell layer (shaded gray). Distortion is a result of histologic processing. Scale 0.5 mm **(C)** TDT arrays in the dorsal hippocampus were localized by tracking their passage (white arrows) and endpoints (red arrows). Note the axons from the MS (green) expressing ChR2-EYFP surrounding the pyramidal cell layer (yellow arrow) and the electrode array. Scale 200 mm.

Channelrhodopsin-2 expression in the MS (**Figure 2A**, green) and hippocampus (**Figure 2B**, red) was robust upon histologic evaluation. From the MS, axonal projections to the hippocampus (**Figures 2A, C**) were readily apparent, coinciding with the passage of the electrodes (**Figure 2C**, red and white arrows) and the hippocampal pyramidal cell layer (yellow arrow). The NeuroNexus array also passed alongside the expressing pyramidal cell layer of the hippocampus (**Figure 2B**). Consequently, we would expect our recordings to appropriately reflect the influence of optogenetic stimulation on these respective neuron populations.

### VALIDATION OF HIPPOCAMPAL RESPONSE TO PULSATILE STIMULATION PATTERNS IN THE MEDIAL SEPTUM

**FIGURE 3:**
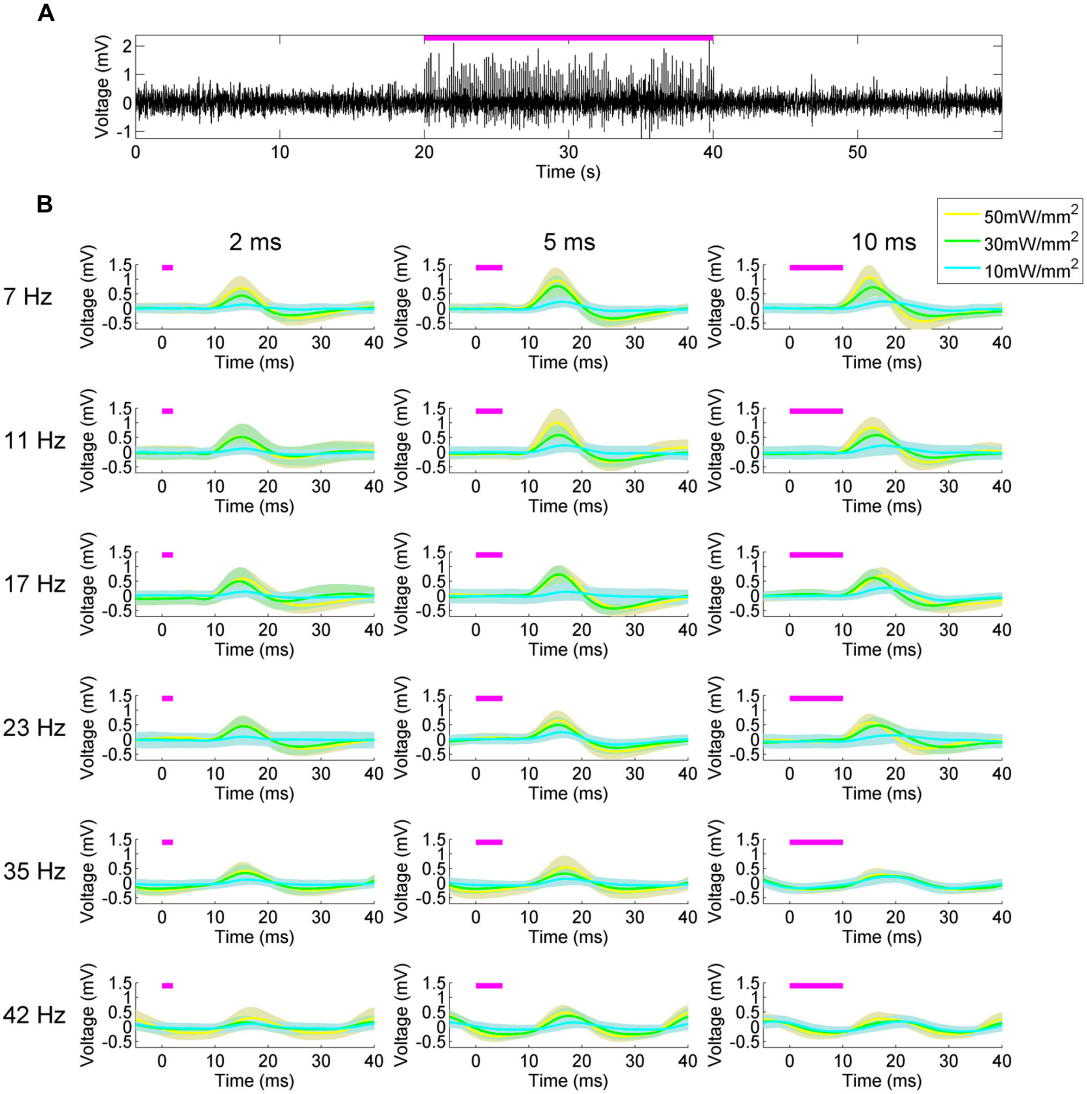
**Peristimulus average hippocampal LFP responses to medial septal stimulation reveal the influence of stimulation parameters on waveform shape. (A)** Hippocampal LFP response to 50 mW/mm^2^, 7 Hz, 10 ms square-wave optical stimulation of the MS (magenta bar). **(B)** Responses to stimuli at different frequency (rows), pulse width (columns), and intensities (blue, green, yellow, respectively). Lines indicate the mean response and the shaded areas indicate the SD.

To validate the effectiveness of the platform, we first explored the LFP response in the dorsal hippocampus to square-wave pulsatile stimulation of the MS (**Figure 3**). The MS has been stimulated electrically previously, producing a stimulus-frequency specific response (McNaughton et al., 2006) that we hypothesized we would recapitulate. At 50 mW/mm^2^, stimulation of the MS produced readily visible delayed pulsatile responses in the hippocampal LFP in both the CA1 and CA3 layers during the stimulation epoch (**Figures 1B and 3A**). These responses did not persist into the post-stimulation epoch, but instead were highly time-locked to the stimulus onset and offset. In order to examine the waveform of the LFP response, a peristimulus average was constructed by determining the mean LFP signal between 5 ms preceding and 40 ms following onset of each stimulus pulse. These were calculated across every stimulation parameter to produce the mean (solid line) and SD (shaded area; **Figure 3B**). As expected, the stimulation parameter specifications had a large impact on response waveform amplitude, shape, and timing. Increasing the amplitude of the stimulus pulse tended to generate a quicker time to peak response. Intriguingly, while increasing the pulse width at lower frequencies increased the amplitude of the response, at higher frequencies (23+ Hz) this was not the case. Indeed, the response to a 35 or 42 Hz, 10 ms stimulus looked remarkably similar regardless of stimulation intensities, with the primary differences manifesting in phase. Biphasic responses were also noted at higher intensities and lower frequencies, whereas unipolar depolarization was most common at 10 mW/mm^2^. At frequencies greater than 35 Hz, the response waveform became largely sinusoidal.

**FIGURE 4:**
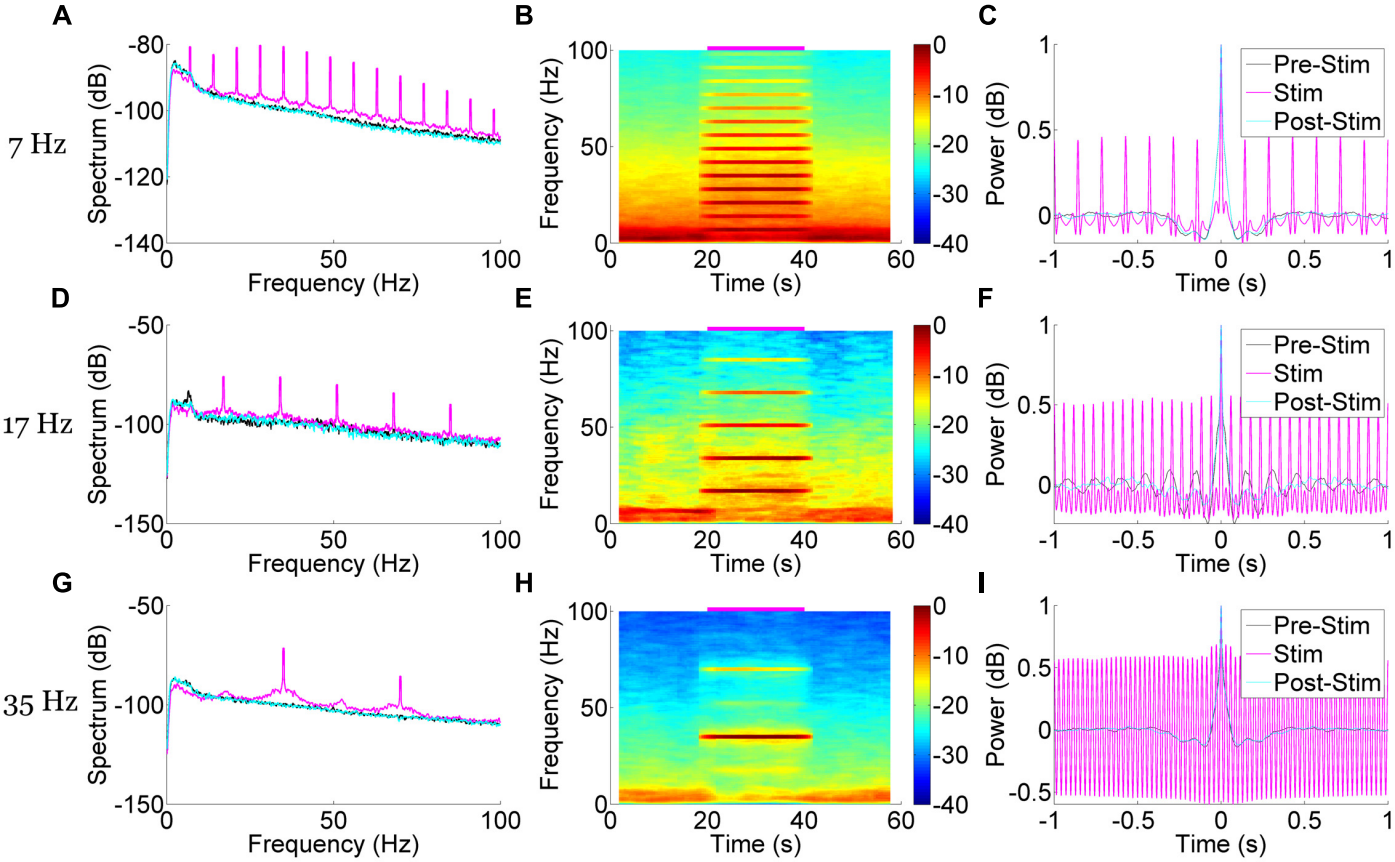
**Spectral and correlational response to medial septal pulse stimulation demonstrate time-locked and frequency specific responses.** Stimulation at 50 mW/mm^2^, 10 ms pulse width, and 7 Hz **(A–C)**, 17 Hz **(D–F)**, and 35 Hz **(G–I)** each produced unique and frequency-specific responses in the spectrum **(A,D,G)**, spectrogram **(B,E,H)**, and autocorrelation **(C,F,I)** of the LFP signal time-locked to the stimulation. Blurred edges near stimulus onset and offset are a result of a wide (4 s) moving window that better resolved the frequency-specificity of the response. The autocorrelation demonstrates a highly correlated LFP signal at stimulation time points, suggesting a locking of oscillatory phase to the stimulus.

To further characterize the hippocampal LFP response to pulsatile stimulation, we examined the spectral properties of the mean signal from six trials of 50 mW/mm^2^, 10 ms stimulation pulses at 7, 23, and 35 Hz (**Figure 4**). In all cases, multitaper spectrograms were generated using seven tapers (*T* = 4 *W* = 1) and a 4 s long moving window iterating at 0.5 s. This wide temporal window resulted in some temporal blurring of the stimulation onset and offset into the non-stimulation epochs, but allowed us to more precisely resolve the frequency domain. A clear increase in power in the spectrum corresponding to the stimulation frequency was apparent during the stimulation epoch as compared to the pre- and post-stimulus epochs in all cases (**Figures 4A,D,G**). A spectrogram of each case revealed the temporal precision of this response (**Figures 4B,E,H**), as well as some of the interactions with power at other frequencies. In all cases low-frequency (1–10 Hz) power was reduced as compared to the pre- and post-stimulus epochs, presumably via stimulation-controlled hijacking of the LFP signal. Examining the mean autocorrelation lends further support to this idea: during stimulation in all cases, the signal became highly correlated at stimulation frequencies (**Figures 4C,F,I**). At higher frequencies the oscillatory nature of the LFP response dominated (**Figure 3B**), resulting in a highly correlated and almost sinusoidal signal that indicated the LFP rhythm was largely dominated and locked to the stimulus frequency and phase.

**FIGURE 5:**
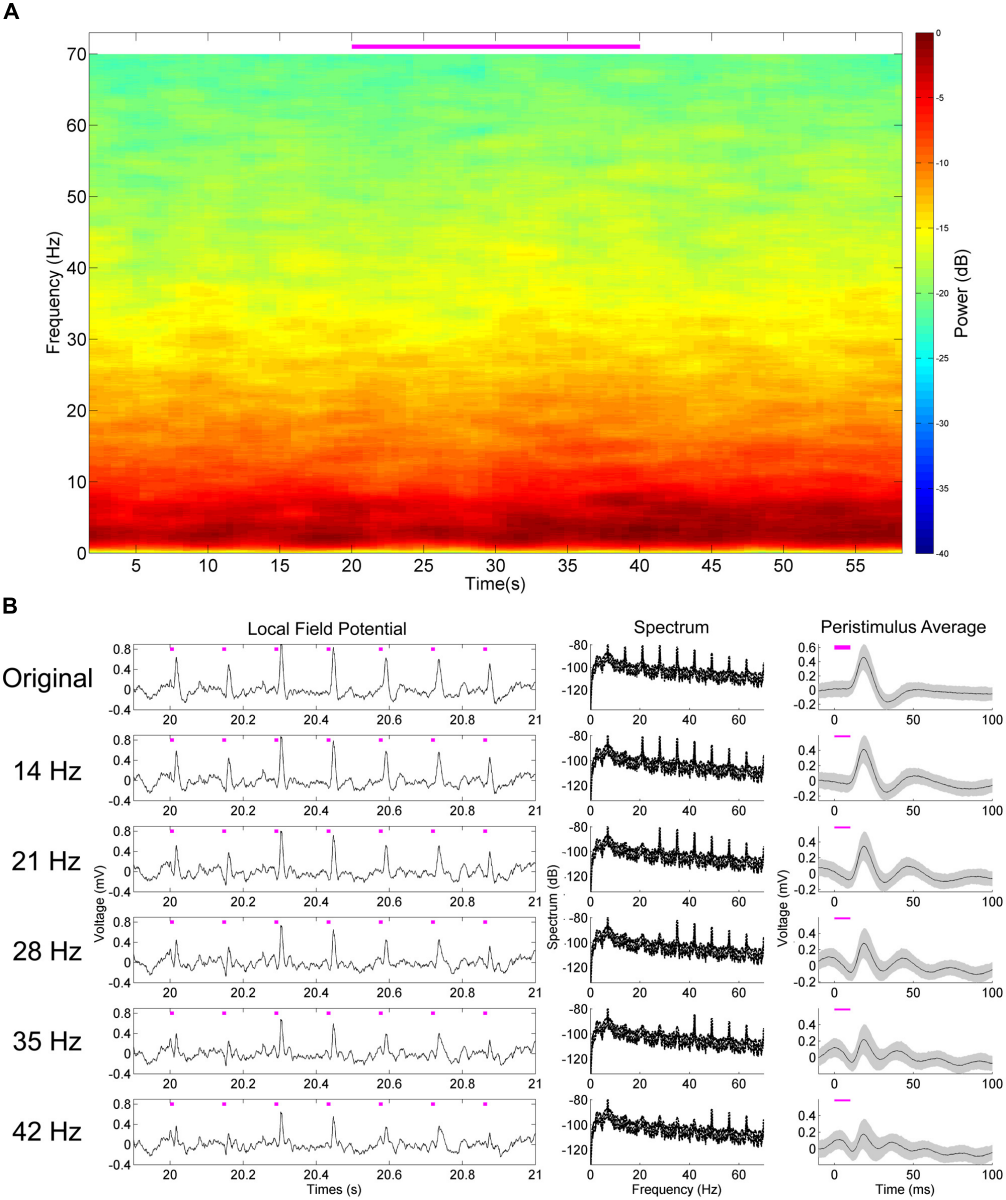
**Harmonic deconstruction demonstrates their participation in non-oscillatory dynamics of the hippocampal pulse response to medial septal stimulation.** Harmonics and artifacts of stimulation are not present in control subjects **(A)**. Successively removing the harmonics noted in the experimental animal (14, 21, 28, 35, and 42 Hz) results in an increasingly sinusoidal response waveform – as demonstrated in the peristimulus average **(B)**. The harmonics are consequently necessary for the non-sinusoidal aspects of the original response waveform.

Aside from increases in power at the stimulation frequency, there were concomitant increases of power at harmonics of that frequency. In the case of 7 Hz stimulation, power was also increased at 14 Hz, 21 Hz, and so forth (**Figures 4A,B**). These harmonics were neuronal in origin and a part of the response signal, as they did not arise when we stimulated an AAV5-hSynapsin-EYFP control animal (**Figure 5A**). It should also be noted that the physical distance between the ferrule and the electrode array (>2 mm) suggests against any photoelectric artifacts, due to the propensity of light to scatter in neural tissue (Adamantidis et al., 2007). However, we suspected that these were a result of the Fourier decomposition of the response waveform, rather than originating in a separate neuronal process or response. In order to distinguish the roles these harmonics play in the signal compared to the primary response at the stimulation frequency, we systematically removed the harmonics from the LFP (rmlinesc.m, Chronux; Bokil et al., 2010). With this algorithm, the time-series signal is converted to frequency space, and then the spectrum is interpolated across at the defined frequencies, removing significant sine waves from continuously recorded data without altering phase properties – as would occur with a notch filter. This has been used previously to remove the line noise resulting from nearby electronics and power sources (Viswanathan and Freeman, 2007). As we progressively removed harmonics from the LFP response to 50 mW/mm^2^, 7 Hz, 10 ms stimulation, the peristimulus average became increasingly sinusoidal, centered on the stimulus frequency (**Figure 5B**). The harmonics therefore play an integral role in generating the waveform of the LFP pulse response, particularly as the waveform deviates from the pure sinusoid of the stimulation frequency.

**FIGURE 6:**
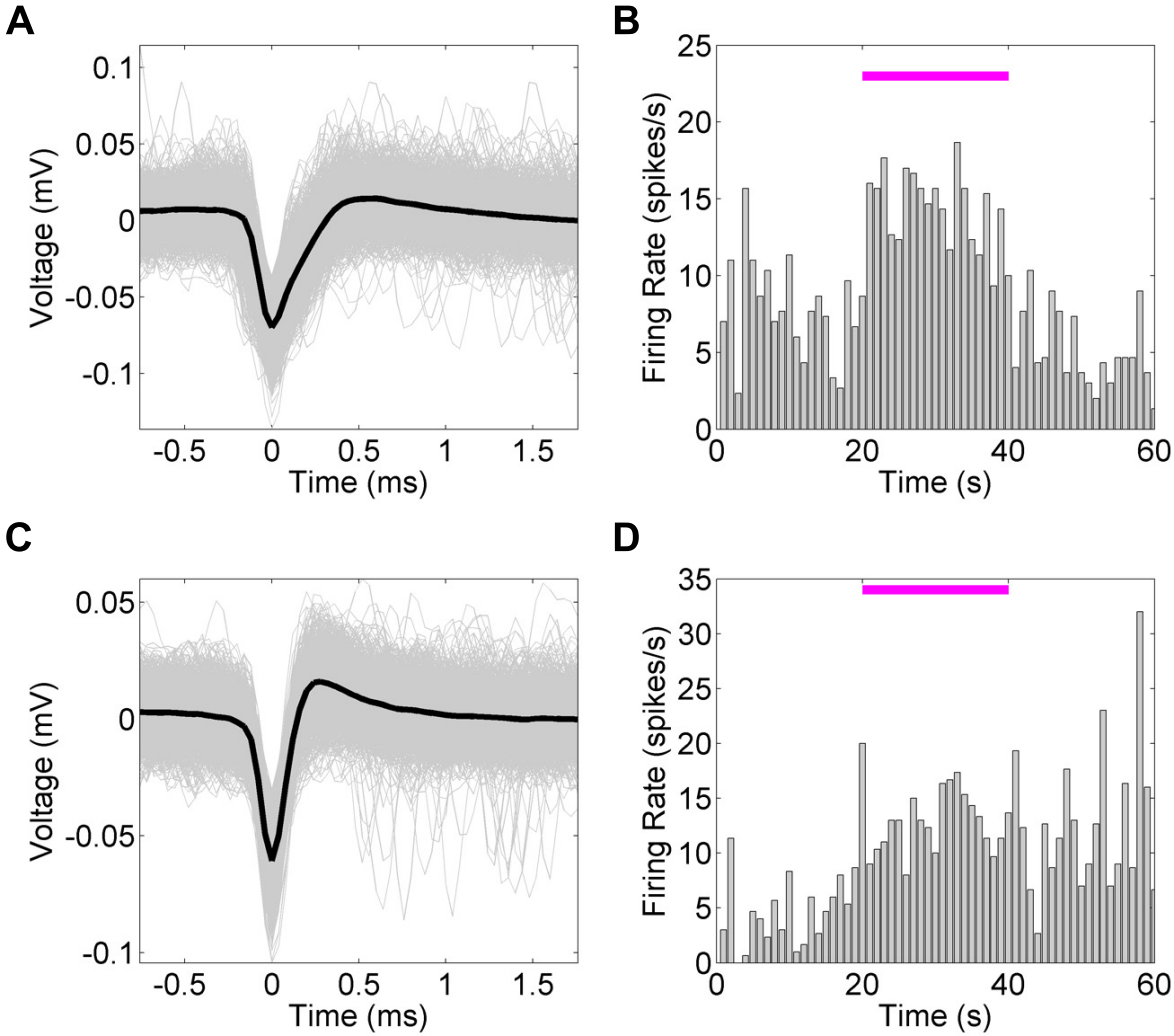
**Hippocampal single unit firing rates increase in response to optical stimulation of the MS.** Mean firing rates for two single units **(A,C)** identified from 50 mW/mm^2^, 23 Hz, 10 ms stimulation trials. Mean firing rate **(B,D)** tended to increase during the stimulation period. In the second case **(C)**, the increase in firing rate remained increased during the post-stimulation epoch **(D)**.

We next examined the system’s ability to detect hippocampal single-unit responses to medial septal optogenetic stimulation (**Figure 6**). NeuroRighter is capable of identifying and sorting units online (Newman et al., 2013). NeuroRighter can also store raw data for oﬄine sorting, however, and so to demonstrate this capability we isolated units oﬄine from 25 kHz sampled data using Matlab scripts combining wavelet transformation and superparamagnetic clustering (wave_clus; Quiroga et al., 2004). Two example units were analyzed for waveform (**Figures 6A,C**) and mean firing rate (**Figures 6B,D**) properties before, during, and after a 50 mW/mm^2^, 23 Hz, 10 ms stimulus train.

In both cases the mean firing rate increased during the stimulation epoch, as calculated across several trials. The firing rate returned to baseline for the first unit (**Figures 6A,B**), whereas the second unit maintained the new average firing rate during the post-stimulus epoch (**Figures 6C,D**). As these are only examples of the capabilities of NeuroRighter to explore single-unit activity, this study was not powered to statistically compare these results, but it was noted that several units demonstrated a trend toward increased firing rate during the stimulation epoch.

### ALTERNATIVE, CUSTOMIZABLE STIMULATION PATTERNS

NeuroRighter is capable of generating complex and customizable stimulation patterns using scripted protocols (Newman et al., 2013). In order to demonstrate examples of this capability, we demonstrate how alternative optical stimulation patterns in the MS could alter hippocampal neural activity in our *in vivo* septohippocampal axis experiments. The results are presented from the combined analysis of several trials.

#### 5 Hz jitter

**FIGURE 7:**
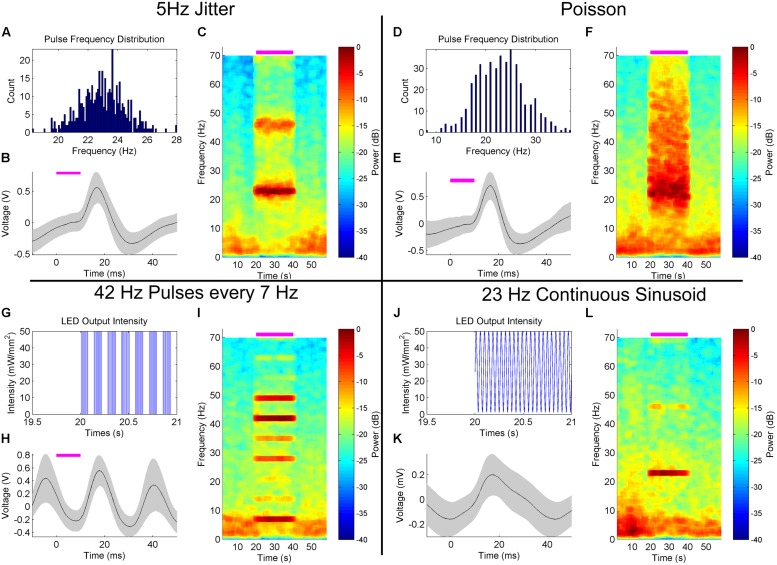
**Hippocampal LFP response to alternative, customizable optical stimulation patterns in the MS. (A–C)** Jittering the frequency of 50 mW/mm^2^, 10 ms stimulation pulses ±5 Hz within a normal distribution centered on 23 Hz **(A)** produced a peristimulus average waveform **(B)** similar to fixed-frequency simulation (**Figure 3**), but with temporal variance in the peak response frequency during stimulation** (C)**. This stimulation reduced the power at frequencies <10 Hz. **(D–F)** Poisson 50 mW/mm^2^ 10 ms pulses generated with a frequency of 23 Hz **(D)** demonstrated a similar LFP peristimulus average response **(E)** and a broadband increase in power that did not influence <10 Hz power **(F)**. **(G–I)** Four 50 mW/mm^2^, 42 Hz, 10 ms pulses generated at a 7 Hz burst frequency **(G)** produced a sinusoidal peristimulus waveform **(H)** similar to constant 42 Hz stimulation (**Figure 3**). Harmonics of the 7 Hz oscillation widely varied in amplitude **(I)**, likely due to constructive and destructive interference between the 42 and 7 Hz stimulation response signals.** (J–L)** Continuous sinusoidal oscillation **(J)** generated a sinusoidal peristimulus average **(K)**, of lower amplitude than pulsed stimulation. Power was concentrated at the stimulus frequency **(L)**, with reduced harmonic power.

In **Figures 4** and **5**, each stimulus pulse occurred at the same frequency during the stimulation epoch, producing a very frequency-specific increase in power in the hippocampal LFP. In the first experiment in alternative stimulation patterns, we introduced a jitter in the interpulse interval based on a random normal distribution of ±5 Hz surrounding the arbitrarily examined stimulus frequency of 23 Hz (**Figure 7A**). The resulting 50 mW/mm^2^, 10 ms pulsed stimulus produced similar depolarization/hyperpolarization responses to that of the fixed-frequency pulsed stimulation, as seen in the peristimulus averages generated (**Figure 7B**), but notable differences were observed spectrographically (**Figure 7C**). First, the response was more broad and effectively tracked the varying stimulation frequency. This is reflective of the neural networks ability to track the variability introduced into to the stimulation signal. This variability may be more reflective of normal neurologic signals, which rarely have the frequency-specificity of artificial stimulation. Note that a stimulation harmonic is also apparent, with similar variability as seen in the primary response signal. The spectrogram also demonstrates an increase in power across frequencies greater than 25 Hz during the stimulation, and a concomitant reduction in power at frequencies less than 10 Hz.

#### Poisson distribution

In our next example experiment, we stimulated the MS with a Poisson distribution of 10 ms pulses at 50 mW/mm^2^, generated at an average frequency of 23 Hz independent of the previous stimuli (**Figure 7D**). A similarly stereotyped peristimulus average response was observed (**Figure 7E**). However, the increase in spectral power was much broader than that generated by fixed or jittered-frequency stimulation (**Figure 7F**). A smear of increased power was observed during the stimulation epoch, extending from ∼15–70 Hz, peaking at the stimulation frequency average of ∼23 Hz. Also observed was a reduced impact on low-frequency (<10 Hz) power as compared to fixed and jittered stimulation pulses.

#### Cross-frequency stimulation

Cross-frequency interactions, such as those between theta and gamma frequencies, are thought to play an important role in neural processing, such as perception and memory (Jensen and Colgin, 2007). In order to try and artificially generate a theta–gamma coupled state, we stimulated the MS at 50 mW/mm^2^ with four 10 ms pulses at 42 Hz with the cycle occurring at a frequency of 7 Hz (**Figure 7G**). This produced a highly sinusoidal pattern in the LFP, as demonstrated by the peristimulus average (**Figure 7H**) and consistent with what has been observed previously (**Figure 3**). Spectral analysis demonstrated a complex response dominated by power bands at 7 and 42 Hz (**Figure 7I**). Harmonics of the 7 Hz response were visible, but the amplitude varied considerably and in a pattern unlike that previously encountered (**Figures 4** and **5**). It is likely that constructive and destructive interference between the harmonics of the 7 and 42 Hz components of the response are responsible for the particular patterning observed.

#### Continuous sinusoidal

Continuous optical stimuli, as opposed to pulsed stimuli, can introduce stimulus currents that better mimic natural synaptic bombardment (Tchumatchenko et al., 2013). Therefore, we also explored stimulating with a continuous 23 Hz sinusoidal signal (**Figure 7J**). The average response was more sinusoidal than fixed frequency (**Figure 7K**). As in other stimulation cases, power was largely concentrated at the stimulus frequency as well, with a reduced harmonic component as compared to the fixed-frequency pulses (**Figure 7L**). Intriguingly, this stimulation pattern seemed to alter the LFP at frequencies other than just the stimulation frequency, with stimulation onset correlating with a consolidation of power at theta frequencies into two discrete bands as calculated across several trials.

### VALIDATION OF HIPPOCAMPAL RESPONSE TO PULSATILE STIMULATION PATTERNS IN THE HIPPOCAMPUS

In our second example experiment, we explored stimulation and recording from the same site, namely, the dorsal hippocampus (**Figure 2B**). NeuroRighter is compatible with a wide variety of electrode configurations, as evidenced in our use of the combined NeuroNexus array and optical ferrule in this example (**Figure 1J**). Optically stimulating and electrically recording in the same location does possess a significant caveat, in the form of optically induced artifacts on the recording electrodes (Ayling et al., 2009; Han et al., 2009; Cardin et al., 2010) that must be separated from the true neurologic signal. This has long been a problem with electrical stimulation and recording, where the multi-fold difference between the stimulation and recording regimes readily obscures or saturates the signal (Wagenaar and Potter, 2002; Rolston et al., 2010a). The photoelectrochemical artifact, or Becquerel effect (Khurram and Seymour, 2013), is not of the same magnitude; it is typically on the same order as the electrophysiologic signal. However, these artifacts still pose a potential problem – can they be separated from the underlying neural signal in order to resolve the LFP and single-unit responses to optical stimulation?

**FIGURE 8:**
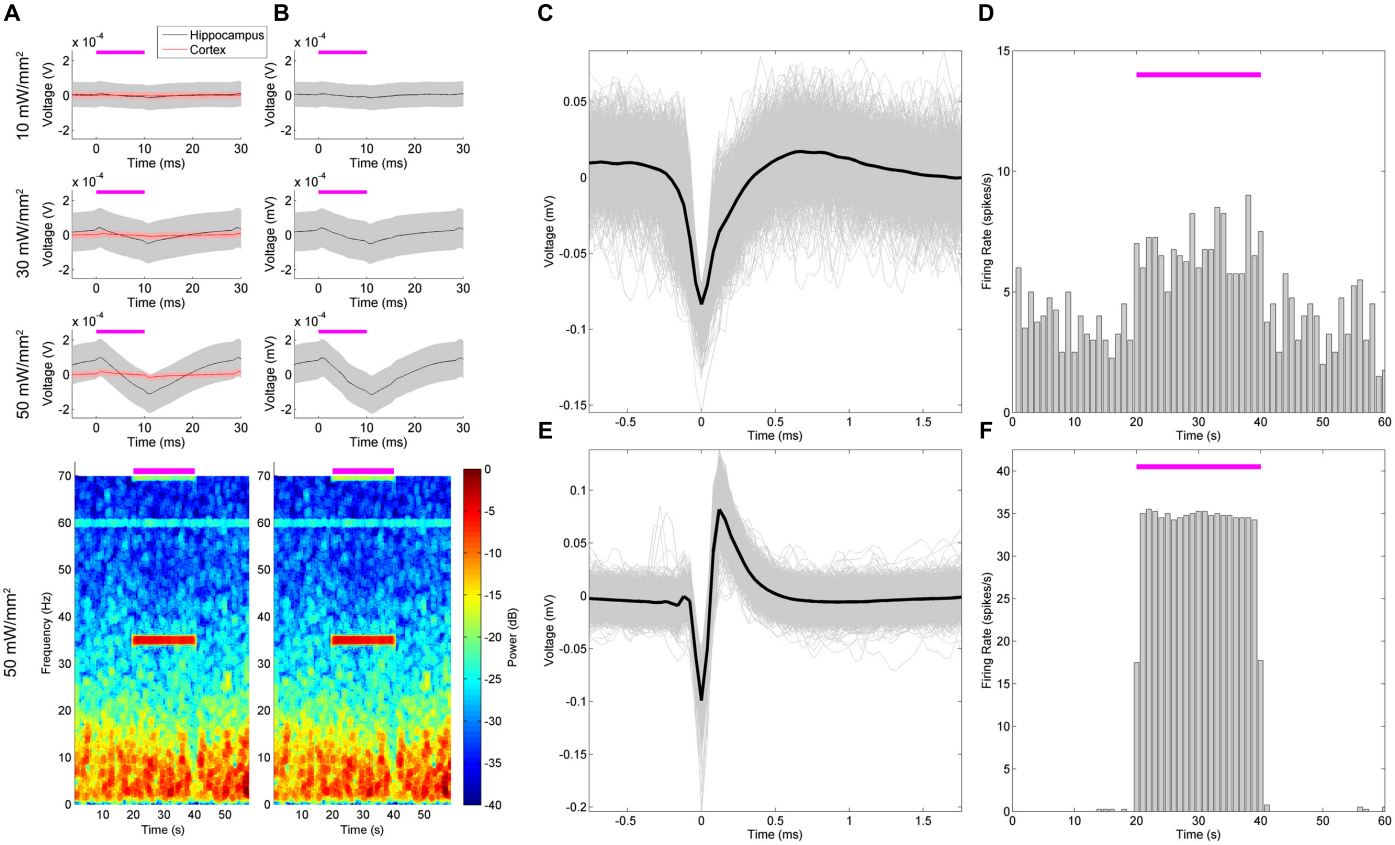
**Stimulation and recording within the hippocampus with a combined NeuroNexus array and ferrule produced a neurologic response and stimulation artifacts.** The dorsal hippocampus was stimulated with a combined array and ferrule (**Figure 1J**) with 35 Hz, 10 ms pulses at 10, 30, and 50 mW/mm^2^. Artifacts of stimulation ( **A**, red) that were intensity-dependent were characterized in the cortex during implantation. The peristimulus average of the hippocampal LFP ( **A**, top) and spectrogram ( **A**, bottom) thus reflect a combination of a neural response and stimulation artifact. We removed the mean artifact from each stimulation time point **(B)**, which revealed the effect of the artifact was negligible relative to the neural response. An example single unit **(C)** increased in firing rate during stimulation **(D)**, and returned to basal firing rate post-stimulus. In contrast, **(E)** demonstrates an artifact from the stimulation signal that resembles a single unit waveform, whose “firing rate” was locked to the stimulation frequency **(F)**.

We first set out to characterize the artifact *in vivo*, and then to separate the artifact from the underlying electrophysiologic signals (**Figure 8**). Stimulating in non-ChR2-expressing cortical tissue, we were able to define the stereotypical artifact waveform at 10, 30, and 50 mW/mm^2^, which appeared in the LFP as charge/discharge depolarization/hyperpolarizations at the beginning and end of the stimulus pulses (**Figure 8A**, red). We did not note DC offsets as seen by Cardin et al. (2010), perhaps due to our particular ground and reference configurations. The electrodes also possessed an iridium oxide coating, as this had been indicated by NeuroNexus Tech (personal communication) to potentially reduce optically induced artifacts. Note that as the intensity increased, so too did the artifact amplitude, but otherwise the waveform was largely stereotyped in appearance. The immediacy, with which these artifacts appeared, as well as the steps we took to prevent optically induced artifacts, suggests that they were actually a result of direct electrical coupling. Since these were unobserved on the TDT microwire arrays and the impedance values between the arrays were similar, we suspect that they resulted from the 21 mm ribbon cable attaching the electrode shank to the Omnetics connector. The cable could be acting as an antenna, picking up the driving current to the LED, and amplifying this noise alongside the neurologic signal.

In the ChR2-expressing regions of the LFP of the dorsal hippocampus (**Figure 8A**, gray), a delayed LFP response to the stimulation was apparent along with the artifact, peaking approximately 11 ms after stimulus onset. Note that this LFP waveform response was only observed in the ChR2-expressing hippocampus (gray) not in the cortex (red). Similarly to medial septal stimulation (**Figure 4**), these responses generated an increase in LFP power at the stimulation frequency (**Figure 8A**, bottom). However, the artifact is still present in the recorded signal. Of note, the artifact, based on its properties in the cortex, is of much smaller amplitude than the neural response. While it could be ignored, it would be unclear whether the changes in spectral power were resulting from the artifact, or the electrophysiological response. Isolating and removing the artifact, therefore, would better reflect the neural response to stimulation.

In order to remove the artifact, we assumed that the artifact would not significantly change between non-expressing tissue and expressing tissue. The distance between the ferrule and the electrodes was fixed during construction (**Figures 1J,K**), and assuming the light-scattering properties of cortical and hippocampal tissue are similar, photo-induced artifacts would largely be the same within the two regions. Furthermore, electrical coupling between the ribbon cable and the LED stimulation input signal would not be expected to differ between the cortex and hippocampus. Thus, to remove the artifact signal oﬄine, we subtracted the mean artifact recorded in the cortex – where there was no ChR2 expression – from the LFP recording in the hippocampus (**Figure 8B**). As the neurophysiologic response was much larger amplitude than the artifact, little appreciable change in spectrographic power was noted (**Figure 8B**, bottom).

While the artifacts in the LFP were readily identifiable from the underlying neurophysiologic signal, the single-unit responses proved difficult to resolve. While common median referencing was employed to attempt to improve the signal to noise ratio of the action potentials (Rolston et al., 2009a), it remained difficult to distinguish true single-units from artifacts. This is demonstrated in (**Figures 8C–F**), wherein a unit believed to be real, and a unit believed to be an artifactual response, are presented. The first detected unit (**Figures 8C,D**) had a basal firing rate preceding the stimulus that increased during the stimulation epoch in successive trials. The second detected unit (**Figures 8E,F**) also increased its firing rate during the stimulus, and appeared to be largely locked to stimulus onset. However, the latter unit failed to be detected outside of the stimulation epoch, and despite the favorable appearance of its waveform, appeared to have been consequent to high-pass filtering of the stimulation artifact on this electrode. Without an accompanying intracellular waveform, or a tetrode-based identification scheme, it remains very difficult to clearly define a unit in this fashion. This is particularly a problem if the unit only appears during stimulation, and is locked to the stimulation frequency.

### CLOSED-LOOP STIMULATION

We used NeuroRighter for closed-loop stimulation of MS in which the hippocampal theta-rhythm was used as a control signal to trigger the stimulation of the MS. The control system was implemented using a dynamic link library (DLL) based on the NeuroRighter application programming interface (API; Newman et al., 2013). The API contains a set of tools for interacting with NeuroRighter’s input and output streams. In this framework the DLL accesses the NeuroRighter data servers, performs computation on the neural data, and then generates and introduces stimulation protocols into the stimulation servers, enabling real-time and closed-loop functionality. Latency is largely determined by the called hardware and software – NeuroRighter’s double-buffered StimSrv output had a response latency of 46.9 ± 3.1 ms – but this was reducible to 7–9 ms with alternative triggers, stimulation hardware, and less-complex outputs (Newman et al., 2013). Our implementation made use of StimSrv, which we found to be fast enough for most of our closed-loop requirements, and nicely integrated with the existing LFP data stream without significant hardware or software complexity^4^.

**FIGURE 9:**
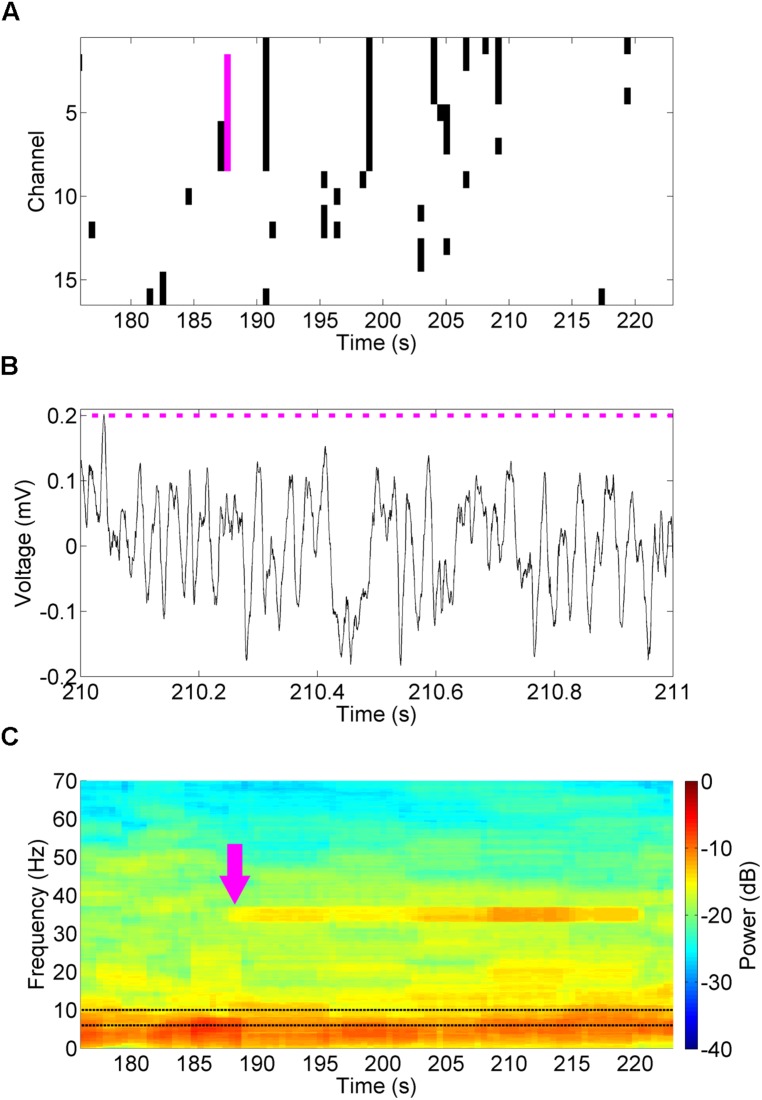
**Closed-loop stimulation of the MS in response to decreased theta power.** A closed-loop DLL program examined theta power (6–10 Hz, **C**, black dotted lines) for decreases in theta power below 3.4% of normal (**A**, black). When this occurred on four or more channels (magenta bar), it triggered a 30 s 50 mW/mm^2^, 35 Hz, 10 ms stimulus in the MS. These pulses (**B**, magenta bars) drove a 35 Hz oscillation in the hippocampal CA3 LFP **(B)** that was readily identifiable in the spectrogram (**C**, magenta arrow).

The LFPs from the 16 channel microelectrode array were sampled by the API and analyzed in this fashion to estimate the power spectral density of theta oscillations (6–10 Hz, **Figure 9A**) over time, relative to the total power of the signal in each time window. The power spectral density was estimated using the signal processing libraries of the Accord.net framework; an open-source framework for building machine learning and signal processing applications. When the normalized theta power dropped below a defined threshold (3.4%) on four or more channels a predefined stimulation profile (50 mW/mm^2^, 35 Hz, 10 ms for 30 s) was generated and sent to the NeuroRighter stimulation servers. These stimulation parameters were chosen for their ease of spectrographic identification, rather than the neurologic or waveform properties. The stimulation parameters and threshold can be adjusted in run-time through a graphical user interface. This arbitrarily designed example closed-loop experiment was effective in generating readily identifiable 35 Hz oscillations in the hippocampal CA3 LFP (**Figure 9B**), also demonstrated as increase in power at 35 Hz in the spectrogram following detection (**Figure 9C**, magenta arrow). Note that during the stimulation the DLL ignored all low-power theta detections, instead stimulating for a predefined period and pattern.

## DISCUSSION

NeuroRighter has been demonstrated to be an adept and versatile platform for real-time, *in vivo* awake and behaving experiments with optogenetic neuromodulation and electrophysiologic recordings. It is capable of open- and closed-loop optical stimulation in a wide variety of user-defined patterns, and provides single-unit and LFP outputs, which are easily and readily analyzed. Through our proof-of-concept experiments and analyses we have demonstrated the capabilities of this system, its potential application in several different custom experimental paradigms, and suggest future endeavors that are worthy of exploration.

As we suspected, the parameters of square-wave optical stimulation in our medial septal stimulation experiments had a significant impact on response waveform properties (**Figure 3**). As we are stimulating in the MS and recording in the hippocampus, the LFP responses we detected were likely the result of post-synaptic potentials generated via medial septal axons (Buzsáki et al., 2012). At higher stimulation frequencies the response became increasingly sinusoidal and decreased in amplitude. There has long been evidence that ChR-2-infected neurons have difficulty following stimulation patterns at >40 Hz (Yizhar et al., 2011). A decrease in LFP response amplitude might therefore be assumed at frequencies >40 Hz as a result of less reliable spike generation: fewer neurons are following the stimulus and generating action potentials, so the signal conducted to the hippocampus – manifested in the hippocampal post-synaptic LFP – is reduced. However, the stimulation frequencies we explored are within this experimentally determined acceptable window. We hypothesize instead that the pattern of decreasing amplitude with increasing stimulation frequency is instead a consequence of the photocycle of ChR2. ChR2 is believed to possess a four-stage photocycle consisting of two open states with different ion conductances, and two closed states (Berndt et al., 2010). The first open state, which is triggered by sudden light intensity changes, results in the non-specific conduction of several ionic species. The second open state, which occurs with prolonged illumination, follows the first open state and is associated with a decrease in the total conductance, in part due to increased selectivity for H^+^ ions, as well as the accumulation of channels in non-conducting states. The waveform response properties we observed may then be a result of similar accumulation of ChR2 channels in these non-conducting states, whereas low-frequency stimulation is able to more maximally activate a recycled and conductive population of light-sensitive ion channels. This hypothesis also provides an explanation for the observation that longer pulse widths tended to alter the time-to-peak responses with different intensities. With short pulse widths the primary conductive mediator would be the first, fast open state. With longer pulse widths the second, slower conducting open state could come into play, delaying the time-to-peak with a later contribution to the response waveform. Computer modeling of these dynamics could provide more quantitative hypotheses that would better reveal the influence of stimulation parameters on these responses, as well as greater insight into the ChR2 channel.

The large influence of stimulation parameters on the response waveform in these characterization experiments suggest that care must be taken in experimental design. Intensity will influence the volume of neural tissue activated, as has been modeled (Adamantidis et al., 2007), but the frequency and pulse width of the stimulation may also influence its impact. Longer pulse widths may induce multiple response action potentials, and also provide more time for the light to convert ChR2 channels into the open and conducting state. In our experiments this produced a higher amplitude response in the downstream LFP at frequencies <35 Hz. At higher frequencies the amplitude of the waveform was independent of intensity and the waveform was sinusoidal. The duty cycle and intensity of the stimulus are consequently both highly influence the waveform response, and should be carefully chosen based on the desired output.

In addition, alternative temporal patterns of stimulation can also influence the neural response. Increasingly, alternative stimulation patterns are being explored for use in clinical deep brain stimulation therapies (Brocker et al., 2013). Indeed, the regimented frequency-specificity of our existing therapies and experiments appear quite artificial when compared with the natural oscillations within these neural circuits. Alternative stimulation patterns that better approximate neurologic signals, such as those presented here (**Figure 7**), may prove more effective in eliciting behavioral and experimental outcomes. Normal physiologic rhythms do not tend to have the frequency or phase specificity of artificial stimulation, and more varied stimuli may consequently affect neural networks differently. Poisson stimulation patterns may better reflect the stochastic firing patterns of neurons and in some cases may prove more effective that constant-frequency stimulation (Quinkert et al., 2010; Wyckhuys et al., 2010). Cross-frequency coupling has a role in spatial memory (Shirvalkar et al., 2010), and sinusoidal stimulation could provide less synchronizing input to the neural network.

The artifacts of optical stimulation that we and others have observed (**Figure 8**), while of significantly less magnitude than equivalent electrical stimulation artifacts, do obscure and potentially influence the underlying neurophysiologic activity. In our hands these artifacts have proven very array-dependent, and others have suggested some mechanisms for reducing and removing them (Cardin et al., 2010). As they can prove quite insidious, leading to false detections as single units, robust methods for preventing, defining, and removing such artifacts will be necessary to limit improper conclusions.

The NeuroRighter platform provides a low-cost, open-source, real-time solution for optogenetic neuromodulation and multielectrode electrophysiology in awake and behaving animals. It is readily customizable to a number of applications, including open- and closed-loop experimentation with a variety of stimulation patterns, recording electrodes, and behavioral tasks.

## AUTHOR CONTRIBUTIONS

Nealen G. Laxpati designed hardware adaptations, ferrules, calibration hardware and software, performed the experiments and their analysis. Jonathan P. Newman and Riley Zeller-Townson wrote the adaptations to the NeuroRighter software for open and closed-loop stimulation, and Babak Mahmoudi coded the closed-loop stimulation experiment. Claire-Anne Gutekunst and Nealen G. Laxpati prepared the viral vectors and performed histology. Robert E. Gross oversaw the work and advised platform and experimental design, and data analysis. All authors contributed to the manuscript.

## Conflict of Interest Statement

The authors declare that the research was conducted in the absence of any commercial or financial relationships that could be construed as a potential conflict of interest.

## Footnotes

1 http://code.google.com/p/neurorighter

2 STL and SolidWorks files, as well as the labview ^∗^.vi, are available at https://sites.google.com/site/neurorighter/share

3 STL and SolidWorks files available at https://sites.google.com/site/neurorighter/share

4 The custom dll file is available at https://sites.google.com/site/neurorighter/share
